# Right-Sided Aortic Arch: A Computed Tomography Angiography Investigation, A Systematic Review with Meta-Analysis

**DOI:** 10.3390/jcm13113105

**Published:** 2024-05-25

**Authors:** George Triantafyllou, Savvas Melissanidis, Marianna Vlychou, George Tsakotos, Nikos Pantazis, Katerina Vassiou, Christos Tsiouris, Maria Piagkou

**Affiliations:** 1 Department of Anatomy, School of Medicine, Faculty of Health Sciences, National and Kapodistrian University of Athens, 115 27 Athens, Greece; gtsakotos@gmail.com (G.T.); tb.christos@gmail.com (C.T.); 2 Radiological Clinic, Asklipios MEdica, 581 00 Veroia, Greece; savvasmelissan@gmail.com; 3 Department of Radiology, University Hospital of Larissa, 413 34 Larissa, Greece; mvlychou@med.uth.gr; 4 Department of Hygiene, Epidemiology and Medical Statistics, School of Medicine, National and Kapodistrian, University of Athens, 115 27 Athens, Greece; npantaz@med.uoa.gr; 5 Department of Anatomy, Faculty of Medicine, University of Thessaly, 413 34 Larissa, Greece; avassiou@gmail.com

**Keywords:** aortic arch, variation, right aortic arch, congenital heart diseases, imaging study, meta-analysis

## Abstract

**Background/Objectives**: The right-sided aortic arch (RAA) is an uncommon variation of the aortic arch (AA), characterized by the aorta crossing over the right main bronchus. In the RAA, the descending aorta can be found on either the right or left side of the spine. The current study comprises a comprehensive retrospective computed tomography angiography (CTA) investigation into the prevalence of the RAA within the Greek population. Additionally, we will conduct a systematic review and meta-analysis to elucidate both common and rare morphological variants of the RAA. This research is significant as it sheds light on the prevalence and characteristics of the RAA in a specific population, providing valuable insights for clinical practice. **Methods**: Two hundred CTAs were meticulously investigated for the presence of a RAA. In addition, the PubMed, Google Scholar, and Scopus online databases were thoroughly searched for studies referring to the AA morphology. The R programming language and RStudio were used for the pooled prevalence meta-analysis, while several subgroup analyses were conducted. **Results: Original study**: A unique case of 200 CTAs (0.5%) was identified with an uncommon morphology. The following branches emanated from the RAA under the sequence: the right subclavian artery (RSA), the right common carotid artery (RCCA), the left common carotid artery (LCCA), and the left vertebral artery (LVA) in common origin with the aberrant left subclavian artery (ALSA). The ALSA originated from a diverticulum (of Kommerell) and followed a retroesophageal course. **Systematic Review and Meta-Analysis**: Sixty-two studies (72,187 total cases) met the inclusion criteria. The pooled prevalence of the RAA with a mirror-image morphology was estimated at 0.07%, and the RAA with an ALSA was estimated at <0.01%. **Conclusions:** AA anomalies, specifically the RAA, raise clinical interest due to their coexistence with developmental heart anomalies and possible interventional complications. Congenital heart anomalies, such as the Tetralogy of Fallot and patent foramen ovale, coexisted with RAA mirror-image morphology.

## 1. Introduction

The typical aortic arch (AA) corresponds to a left-sided and three-branched vessel representing the second part of the aorta. It gives off the brachiocephalic trunk (BCT), the left common carotid, and the left subclavian artery (LCCA and LSA). The BCT is divided into the right common carotid and subclavian arteries (RCCA and RSA). The left and right vertebral arteries (LVA and RVA) emanate from the LSA and RSA. The AA anatomy has been extensively studied due to its high clinical implications [1,2,3,4]. Popieluszko et al. [1] first performed a systematic review with a meta-analysis of the most frequent AA variants, and Natsis et al. [2] and Tsiouris et al. [3] reviewed the left-sided aortic arch (LAA) variants in cadaveric and imaging studies, respectively. The difference between these reviews and Popieluszko et al. [1] was that these researchers investigated, except for the most frequent variations [1], even rarer forms of AA morphology [2,3]. Identifying the AA branching pattern is essential to avoid surgical or endovascular complications [1,3]. One of the uncommon AA variants is the right-sided aortic arch (RAA), which was estimated to have a pooled prevalence of 0.2% by Popieluszko et al. [1] during their meta-analysis. The RAA corresponds to the mirror-image of the typical LAA, with the following branch sequence: the RSA, the RCCA, and a common trunk of the LCCA and the LSA. The aorta crossing over the right main bronchus defines the RAA. In the RAA, the descending aorta can be found on either the right or left side of the spine [5]. Acar et al. [4] identified another type of a four-branched RAA with the following sequence: the RSA, the RCCA, the LCCA, and the LSA with an aberrant (ALSA) of a retroesophageal (or not) course, that sometimes coexist with a diverticulum (of Kommerell). If an ALSA is present, the ductus arteriosus originates from this SCA, which leads to an outpouching of the aorta (diverticulum of Kommerell) [6]. Acar et al. [4] added another RAA morphological type, in which there was direct communication between the LSA and the left pulmonary artery. The current study aims to investigate the RAA in a retrospective computed tomography angiography (CTA) study of a Greek population and to present, in a systematic review with a meta-analysis of the RAA, the most standard and rarest morphological variants’ prevalence.

## 2. Materials and Methods

### 2.1. Original Study

Archived computed tomography angiography (CTA) on a 128-slice multi-slice CT scanner was documented at the Radiology Department of Larissa University Hospital. Two hundred CTAs from 142 male and 58 female subjects with a mean age of 62.85 ± 14.08 years were randomly selected and searched for RAA morphology. The CTAs were performed between 2018 and 2022. The exclusion criteria were any pathology that affected the arterial anatomy and low-quality or incomplete scans. The Ethics committee of the University Hospital of Larissa approved the study. The Helsinki Declaration (1964), a set of ethical principles regarding human tissues and research, was considered.

### 2.2. Search Strategy—Selection Criteria for the Systematic Review Study

The study was performed according to the PRISMA (preferred reporting items for systematic reviews and meta-analyses) guidelines for reporting systematic reviews with meta-analyses [7]. Articles were found by conducting systematic searches in the PubMed, Google Scholar, and Scopus online databases using the keywords “aortic arch”, “right-sided aortic arch”, “right aortic arch”, “variations”, “aortic arch variations”, “right-sided aortic arch variations”, and “right aortic arch variations” in various combinations. Every study that presented AA variations was included until February 2024. Case reports, conference abstracts, and letters to the editor were excluded. The following parameters were extracted: publication year, country (classified on continents), study type (cadaveric, imaging), total sample, and RAA prevalence (classified into types). The mirror-image RAA was classified as Type 1, and the RAA with an ALSA was classified as Type 2.

### 2.3. Statistical Analysis

The statistical analysis utilized the open-source R programming language (R Core Team, 2021) and RStudio software version 4.3.2 (RStudio Team, 2022), along with the “meta” and “metafor” packages [8,9]. The pooled prevalence was calculated using the inverse variance method and the random effects model. The proportions (prevalence) meta-analysis involved the Freeman–Tukey double arcsine transformation, the DerSimonian–Laird estimator for the between-study variance tau^2^ and the Jackson method for a confidence interval of tau^2^ and tau. Cochran’s Q statistic was used to evaluate the heterogeneity across studies and the Higgins I^2^ statistic for quantifying heterogeneity. An I^2^ value of 25–50%, 50–75%, and >75% indicate low, moderate, and high heterogeneity [10,11]. To evaluate the presence of the small-study effect, a DOI plot was created, and the asymmetry of each plot was calculated by the LFK index [12]. Subgroup analyses were performed to estimate the impact of the study’s design (cadaveric, imaging), subjects’ geographical region (continent of origin), and sample size (0–99, 100–299, 300–999, and over 1000 subjects) on the pooled estimation. A *p*-value < 0.05 was set as statistically significant.

## 3. Results

### 3.1. Original Study Results

**Figure 1:**
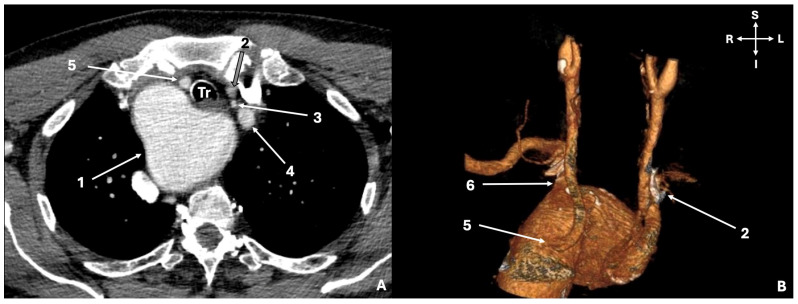
The right-sided aortic arch (RAA). (**A**). Typical computed tomography scan, (**B**). Three-dimension reconstruction. 1—RAA with Kommerell’s diverticulum compressing the esophagus and the trachea (Tr), 2—Left common carotid artery (LCCA), 3—Left vertebral artery (LVA), 4—Left subclavian artery (LSA) with aberrant origin from the diverticulum, 5—Right common carotid artery (RCCA), 6—Right subclavian artery (RSA). S—superior, I—inferior, L—left, and R—right.

A unique case of an RAA was identified out of the two hundred investigated AAs (0.5%; 1/200). This case could not be classified into the two RAA subtypes (mirror-image or ALSA). The following vessels were emanating from the RAA in sequence: the common origin of the LCCA and LVA (LCCA-LVA), the ALSA (originating from Kommerell’s diverticulum and retroesophageal course), the RCCA, and the RSA (Figure 1).

### 3.2. Systematic Review Results

**Figure 2:**
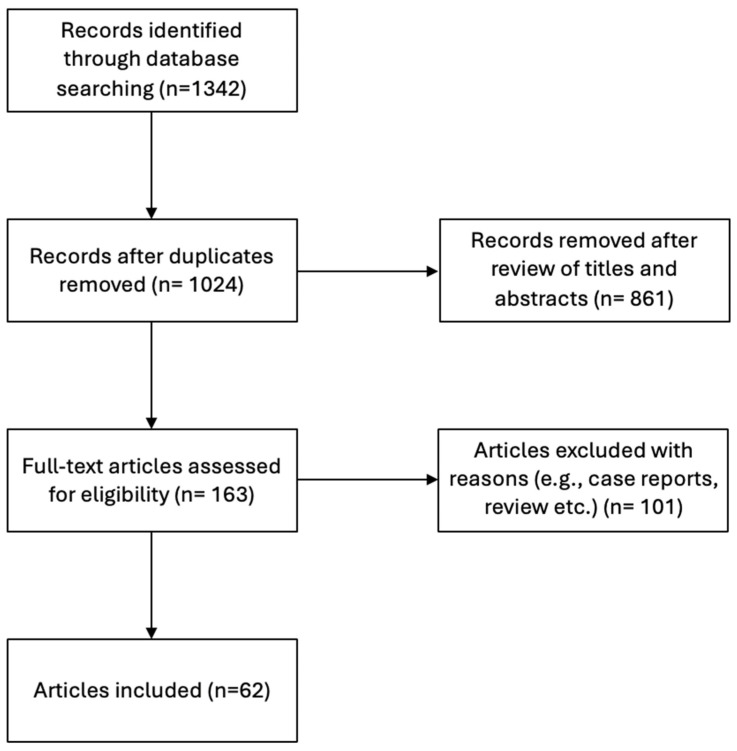
Flow chart of the right aortic arch (RAA) literature search.

The literature search revealed 62 studies meeting the current inclusion criteria, with 72,187 total cases [4,13,14,15,16,17,18,19,20,21,22,23,24,25,26,27,28,29,30,31,32,33,34,35,36,37,38,39,40,41,42,43,44,45,46,47,48,49,50,51,52,53,54,55,56,57,58,59,60,61,62,63,64,65,66,67]. The flow chart is summarized in Figure 2. Based on the sample size, the studies were classified into the following four groups: 0–99 cases (18 studies), 100–299 cases (12 studies), 300–999 cases (16 studies) and 1000 and above (16 studies). These numbers were obtained to distribute the studies equally between four groups and better analyze them in the following sample-size analysis.

### 3.3. Statistical Results

**Figure 3:**
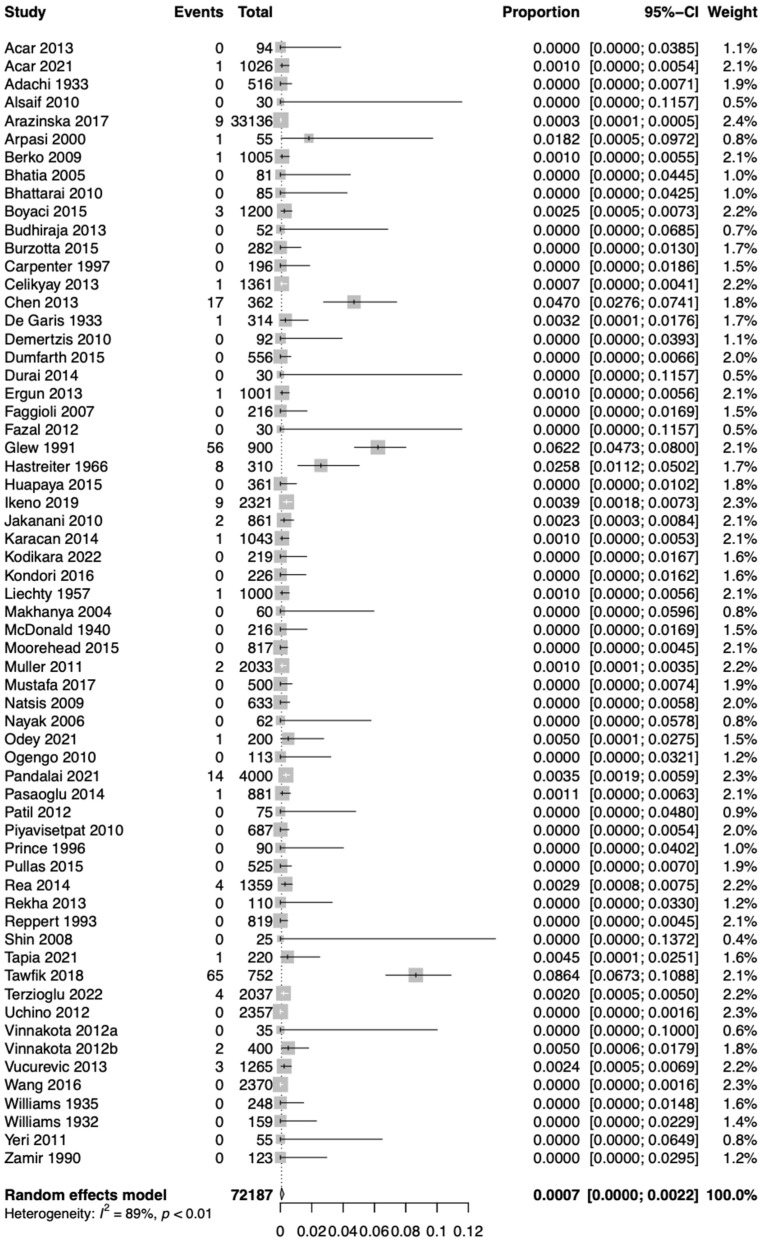
Forest plot showing estimated prevalence of right aortic arch (RAA) Type 1 (mirror-image).

Based on the k = 62 studies (overall cases = 72,187), using the random effect model, the pooled prevalence of Type 1 RAA was estimated as 0.0007 [0.0000; 0.0022]. Therefore, approximately 0.07% of the population is expected to have Type 1 RAA (Figure 3). The estimated heterogeneity was statistically significant (*p* < 0.01) based on the Q test statistic and high degree based on the I^2^ statistic (I^2^ = 89%). 

**Figure 4:**
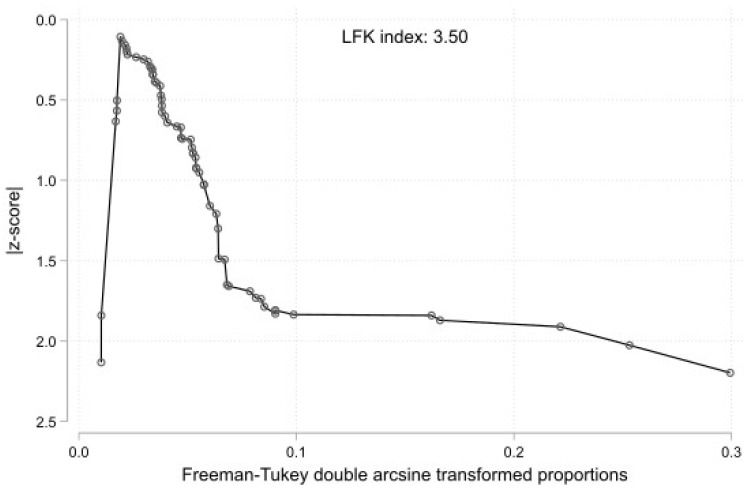
DOI plot with LFK index showing estimated small study effect of right aortic arch (RAA) Type 1 (mirror-image).

The results of the subgroup analyses on the effect of the study’s type, subjects’ geographical region, and study sample on the estimated prevalence of RAA Type 1 are summarized in Table 1. The test for subgroup differences with the study’s design (cadaveric imaging) as a categorical predictor was not statistically significant (*p* = 0.1882). Thus, the study’s design is not an essential moderator of the estimated prevalence of the typical morphology. The studies were categorized based on the subjects’ continent of origin to evaluate the geographical region as a possible moderator of the estimated prevalence of RAA Type 1. The test for subgroup differences was not statistically significant (*p* = 0.2989). However, only one study has been included in the Oceania subgroup, and further studies are required to reach the minimum of four studies per subgroup, as Fu et al. [68] suggested for a (categorical) subgroup variable. The studies were subclassified into four barely equal sample-size groups (0–99, 100–299, 300–999, and over 1000 subjects). The subgroup analysis was not statistically significant (*p* = 0.1830). Additionally, the presence of the small-study effect was evaluated. The DOI plot of the RAA Type 1’s prevalence is shown in Figure 4. Based on the LFK index = 3.50, this implies a small-study effect on RAA Type 1’s estimated prevalence and possible publication bias.

**Figure 5:**
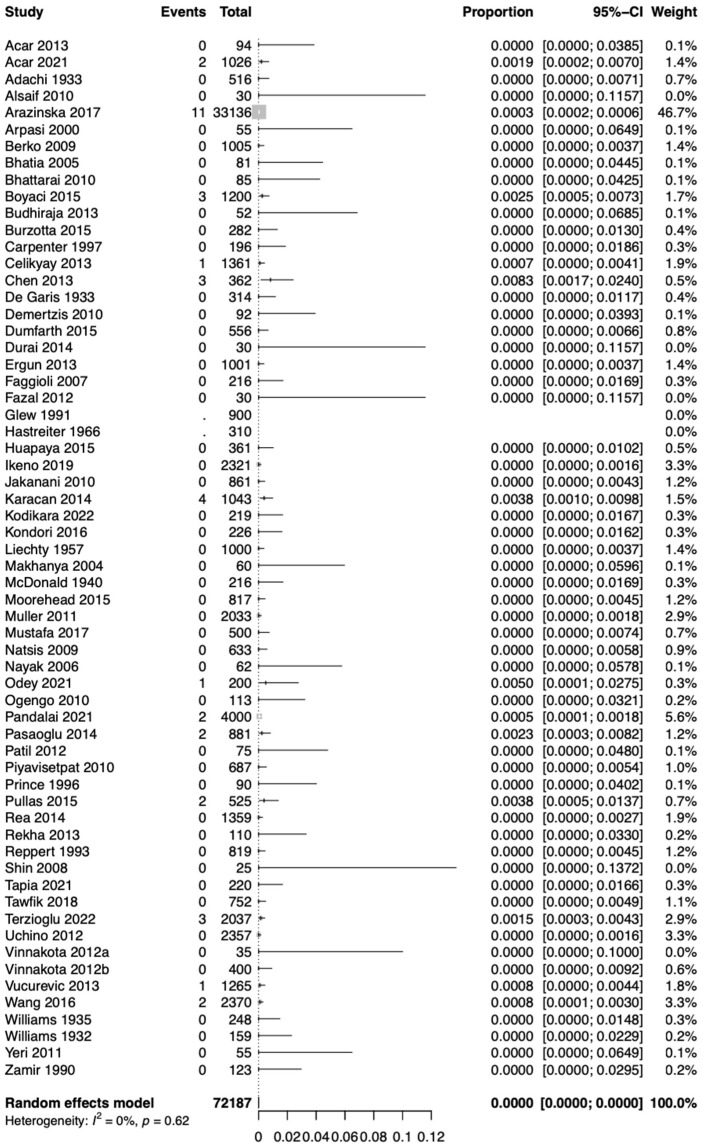
Forest plot showing estimated prevalence of right aortic arch (RAA) Type 2 (aberrant left subclavian artery—ALSA).

Using the random effect model, the pooled prevalence of RAA Type 2 was estimated as 0.0000 [0.0000; 0.0000], and therefore, <0.01% of the population is expected to present with Type 2 RAA (Figure 5). The estimated heterogeneity was not statistically significant (*p* = 0.62) based on the Q test statistic, and low degree based on the I^2^ statistic (I^2^ = 0%). 

**Figure 6:**
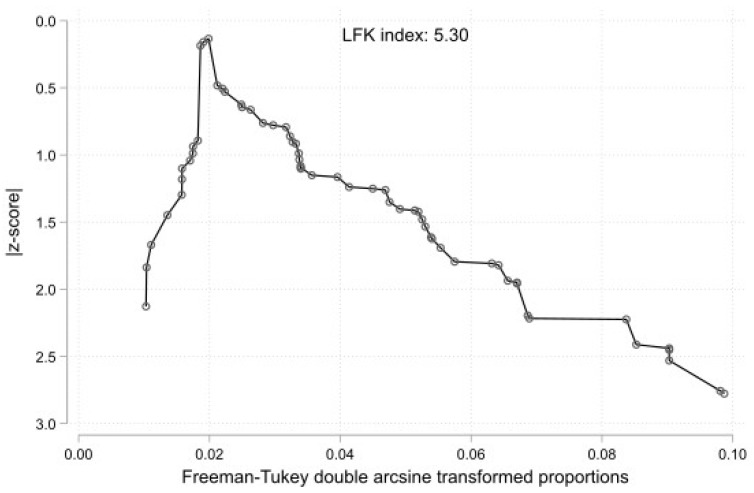
DOI plot with LFK index showing estimated small study effect of right aortic arch (RAA) Type 2 (aberrant left subclavian artery—ALSA).

The results of the subgroup analyses on the effect of the study’s type, subjects’ geographical region, and study sample on the estimated prevalence of RAA Type 2 are summarized in Table 1. The subgroup analysis for the study’s design (cadaveric imaging) was not statistically significant (*p* = 0.5364). In addition, the geographical region was statistically significant (*p* = 0.0462), while only one study was included in the Oceania subgroup, and further studies are required to reach the minimum of four studies [68]. The subgroup analysis between four groups based on sample size studies (0–99, 100–299, 300–999, and over 1000 subjects) was not statistically significant (*p* = 0.1193). The DOI plot of the prevalence of RAA Type 2 is shown in Figure 6. Based on the LFK index = 5.30, this implies a small-study effect on RAA Type 2’s estimated prevalence and possible publication bias.

## 4. Discussion

### 4.1. Embryological Development of the Aortic Arch (AA)

AA development is a very complex procedure; variants most frequently occur in different developmental stages. Rathke proposed the current embryologic model [69]. Six primitive pairs of AAs give rise to the adult LAA. The RAA, which is extensively studied in the present article, probably arises from the fourth pair (of the six primitive AAs). The left fourth primitive AA typically regresses, resulting in the typical LAA morphological anatomy; however, if the left fourth AA persists and the right fourth AA regresses, the RAA will develop [16,69]. It has been proposed that the embryonic blood flow plays a role in differentiating the development process and finally affecting the RAA formation [70]. It is essential to highlight that both arches can persist and create a double AA. 

### 4.2. Morphological Variability of the Left Aortic Arch (LAA)

Before assessing the current study and meta-analysis on the morphology of the RAA, it is essential to address the typical and variant anatomy of the LAA. Popieluszko et al. [1] systematically classified the AA variants. Popieluszko Type 1 corresponded to the typical LAA, with a pooled prevalence of 80.9%. Popieluszko Type 2 is characterized by the misnomer “bovine arch” with the common origin of the RSCA, RCCA, and LCCA (13.6% pooled prevalence). Popieluszko Type 3 was observed when the LVA originated from the AA, with a pooled prevalence of 2.9%. Popieszko Type 2 and 3 combined created the Type 4 (misnomer “bovine arch” along with LVA from AA) with a pooled prevalence of 0.4%. Popieluszko Type 5 corresponded to an AA with the common origin of the two CCA, the so-called bi-carotid trunk, with a pooled prevalence of 0.3%. Popieluszko Type 6 was the AA with an aberrant RSA that was observed with a pooled prevalence of 0.7%. Lastly, Type 7 was characterized as the RAA (0.2% pooled prevalence). After this meta-analysis, Natsis et al. [2] tried to classify AA variants better, adding even rarer types, according to the cadaveric studies. The Natsis proposed classification system consisted of types and subtypes according to the number of branches [2]. Therefore, the typical three branches of AA corresponded to Natsis Type 3b1, with a pooled prevalence of 78%. The most common variation corresponded to the misnomer “bovine arch”, termed brachiocephalic-carotid trunk by Natsis et al. [2]. They observed AAs from one to five branches, with several subtypes in each type [2]. Following Natsis et al.’s [2] classification, Tsiouris et al. [3] applied the same protocol to imaging studies. The most exciting result of the Tsiouris et al. [3] meta-analysis was the statistically significant difference between the number of multidetector computed tomography (MDCT) scanner rows that were used to depict variants. The typical pattern of AA (Natsis Type 3b1 [2]) had a lower prevalence with an MDCT scanner, with less than 64 detected rows. To conclude, Popieluszko et al. [1] were the first to calculate the pooled prevalence of AA variants, while Natsis et al. [2] and Tsiouris et al. [3] provided a more comprehensive way to classify the LAA morphology and also depicted essential results, such as the difference between the MDCT scanner [3].

### 4.3. Morphological Variability of the Right Aortic Arch (RAA)

The RAA morphology is further analyzed from the previously published aortic arch meta-analysis in the current meta-analysis [1]. The RAA of Type 1 (mirror-image) was estimated to have a pooled prevalence of 0.07%, and the RAA of Type 2 (ALSA) was estimated to have a pooled prevalence of <0.01%, implying its rare existence. While performing subgroup analysis for the possible influence of moderators (geographic region, study type, and sample size), the only statistical difference that arose was the geographic region for the RAA of Type 2. The meta-analysis was reinforced with an original anatomical study on 200 CTAs performed on a Greek population that revealed only a unique case of an RAA. Interestingly, this case could not be classified into RAA types of meta-analysis either using Acar et al.’s [4] classification. Therefore, a literature search for rare RAA morphological types (extracted information from case reports) was performed. Inaba et al. [71] reported a case of RAA with the LVA originating from the LCCA, coexisting with an ALSA in a 55-year-old asymptomatic patient. An even rarer RAA form was observed by Tong et al. (2015), who identified a five-branched RAA with the following sequence: a common trunk of LCCA and LVA, RCCA, RVA, RSA, and an ALSA arising from a Kommerell’s diverticulum (which further gave rise to an accessory LVA-ALVA) [72]. Singh et al. [73] presented a rare case of RAA with complete isolation of the left BCT. Isolated LSA has also been observed [74], while Grollman et al. [75] identified a left BCT with a retroesophageal course coexisting with an RAA.

### 4.4. Clinical Implications of the Aortic Arch (AA) Variations

Except for anatomical and embryological interest, AA anomalies, specifically RAA, raise clinical interest due to their coexistence with developmental heart anomalies and possible interventional complications. Congenital heart anomalies, especially cyanotic, such as Tetralogy of Fallot, have been associated with an RAA of Type 1 (which appeared in approximately 75–85% of patients) [76]. The RAA has a distinctively high association with cyanotic congenital heart anomalies. Therefore, cardiac morphology is usually abnormal (Kanne and Godwin, 2010). On the other hand, an RAA of Type 2 is uncommonly related to heart anomalies, except in cases that coexist with the left descending aorta [77]. D’Antonio et al. [78] performed a systematic study with a meta-analysis, examining the coexistence of an RAA with chromosomal anomalies, such as 22q11.2 deletion. They observed that most fetuses with an RAA and normal cardiac anatomy had no associated chromosomal abnormalities, while the risk was approximately 10% [78]. RAA Type 2 (ALSA) has been associated with clinical symptoms due to the ALSA retroesophageal course and possible compression of the trachea, esophagus, and mediastinal structures [16]. The incidence of symptoms increases when an aneurysmal dilatation (Kommerell diverticulum) coexists with the ALSA origin [77]. Faggioni et al. [79] observed that the diverticulum’s walls had a high incidence of medial necrosis; hence, it is prone to dissection and rupture. In the current study, the unique patient with the RAA and the ALSA had this type of diverticulum, similar to the findings of Arazinska et al. [16], who recorded, in all cases of an RAA with ALSA, the coexistence of Kommerell’s diverticulum. However, most anatomical studies did not differentiate cases of an RAA with an ALSA with or without Kommerell’s diverticulum; therefore, we could not differentiate between RAA cases with an ALSA with and without diverticulum. This coexistence, especially in cases of an enlarged diverticulum, may cause a variety of symptoms, such as dysphagia, dyspnea, stridor, and wheezing [16]. Cardiac surgery and endovascular interventions are gold-standard methods to treat AAs and heart pathologies, while interventionists rely on anatomy to perform these procedures [4]. Thus, preoperative imaging techniques, such as CTA with 3D reconstruction, are paramount in accurately depicting cardiac and AA anatomy [3,80].

### 4.5. Limitations

There are a few limitations to be considered within the current study. Firstly, the population sample was limited to Greek patients. Additionally, the CTAs used were based on patients with pre-existing vascular pathology for imaging. Additionally, the study sample was considered low (*n* = 200); however, the study was supplied by a systematic review with a meta-analysis. Nevertheless, the meta-analysis sample had significant heterogeneity; for example, there were studies with different methodologies (clinical and cadaveric studies). Although a statistically significant difference was identified during the geographical subgroup analysis for Type 2 RAAs, it is not appropriate to conclude due to the limited number of studies [68].

## 5. Conclusions

An RAA represents one of the rarest variants of the typical AA, with a pooled prevalence of 0.07%. A quite unusual RAA morphological variant was identified during a retrospective analysis of 200 CTAs. The variant RAA branched into the following sequence: RSA, RCCA, LCCA, and LVA in common origin with an ALSA, which originated from a diverticulum (of Kommerell) and followed a retroesophageal course. Knowledge of AAs has been proven to be of paramount importance for clinicians to avoid pitfalls and iatrogenic lesions.

## Figures and Tables

**Table 1:** Subgroup analysis for right aortic arch (RAA) Type 1 (mirror-image) and Type 2 (aberrant left subclavian artery—ALSA). Statistically significant *p*-values have been highlighted with bold letters.

Morphology	Moderator	Subgroup	Studies’ Number (k=)	Prevalence [95%-CI]	*p*-Value
RAA Type 1	Type of Study	Imaging	42	0.0021 [0.0006; 0.0043]	0.1882
		Cadaveric	20	0.0000 [0.0000; 0.0000]	
RAA Type 1	Nationality	Europe	12	0.0035 [0.0002; 0.0094]	0.2989
		Asia	28	0.0000 [0.0000; 0.0005]	
		America	16	0.0000 [0.0000; 0.0003]	
		Africa	5	0.0085 [0.0000; 0.0554]	
		Oceania	1	0.0000 [0.0000; 0.0211]	
RAA Type 1	Sample Size	0–99 cases	18	0.0000 [0.0000; 0.0015]	0.1830
		100–299 cases	12	0.0004 [0.0000; 0.0022]	
		300–999 cases	16	0.0057 [0.0003; 0.0161]	
		1000 and above cases	16	0.0012 [0.0005; 0.0021]	
RAA Type 2	Type of Study	Imaging	40	0.0000 [0.0000; 0.0001]	0.5364
		Cadaveric	20	0.0000 [0.0000; 0.0000]	
RAA Type 2	Nationality	Europe	10	0.0000 [0.0000; 0.0000]	**0.0462**
		Asia	28	0.0000 [0.0000; 0.0000]	
		America	16	0.0000 [0.0000; 0.0000]	
		Africa	5	0.0000 [0.0000; 0.0011]	
		Oceania	1	0.0000 [0.0000; 0.0211]	
RAA Type 2	Sample Size	0–99 cases	18	0.0000 [0.0000; 0.0010]	0.1193
		100–299 cases	12	0.0000 [0.0000; 0.0012]	
		300–999 cases	14	0.0002 [0.0000; 0.0009]	
		1000 and above cases	16	0.0004 [0.0001; 0.0008]	

## Data Availability

All data are available upon request to corresponding authors.
